# Excitation Energies from the Entanglement Coupled Cluster Model for Doublets

**DOI:** 10.1021/acs.jpca.5c01190

**Published:** 2025-07-21

**Authors:** Sarai Dery Folkestad, Kristine Lauvstad Kruken, Henrik Koch

**Affiliations:** Department of Chemistry, 8018 Norwegian University of Science and Technology, NTNU, Trondheim 7491, Norway

## Abstract

We present a spin-adapted coupled cluster singles and doubles model for the excitation energies of molecular doublets. The entanglement coupled cluster approach represents an unconventional take on the notorious problem of spin adaptation for open-shell species. In this approach, the high-spin open-shell molecular system is coupled to non-interacting bath orbitals to form a total closed-shell system. In entanglement coupled cluster theory, many of the attractive features of the spin-adapted closed-shell coupled cluster are retained: an unambiguous definition of the cluster operator and a terminating Baker–Campbell–Hausdorff expansion. The model produces excitation energies of a quality comparable to that of the closed-shell counterpart. Additionally, some ionized states that cannot be modeled accurately with the alternative equation-of-motion approach for ionized states can be described with the entanglement coupled cluster singles and doubles model.

## Graphical abstract

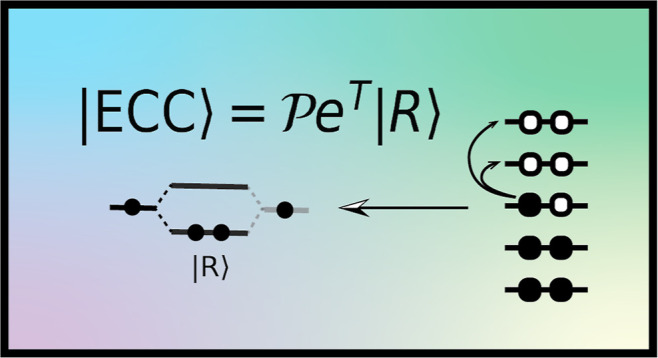

## Introduction

The nonrelativistic molecular electronic Hamiltonian commutes with the spin operators, and the exact electronic wave function is also an eigenfunction of the *S* ^2^ and *S*_*z*_ operators.^1^ In electronic structure theory, a model is spin-adapted if it preserves the spin properties of the exact wave function. While enforcing the wave function to be an eigenfunction of the *S*_*z*_ operator is trivial, satisfying the condition that it also is an eigenfunction of *S* ^2^ may be complicated for spin quantum number *S* > 0, depending on the parametrization of the wave function. Developing a spin-adapted formulation of coupled cluster theory for open-shell species has turned out to be a major challenge.

The unrestricted, or spin–orbital, formulation is an alternative to spin-adapted coupled cluster theory. Unrestricted coupled cluster theory is simple in both its formulation and implementation. However, since the requirement that the wave function is an eigenfunction of the *S* ^2^ operator is lifted, the approach suffers from spin contamination. While the spin contamination of the unrestricted coupled cluster ground state^2^ is limited, even for systems where the UHF spin contamination is significant, the equation-of-motion framework for excited states is highly affected by spin contamination of the reference wave function, and an ROHF reference should be used.^3^ Furthermore, magnetic properties depend explicitly on spin and are likely more sensitive to spin contamination than the energies.

For closed-shell species, the spin-adaptation of coupled cluster theory is straightforward. The cluster operator is defined in terms of singlet (spin-free) excitation operators, which preserve the spin properties of the reference determinant. Since all molecular orbitals are either doubly occupied or vacant, all terms in the cluster operator commute, and the Baker–Campbell–Hausdorff expansion of the similarity-transformed Hamiltonian terminates after four nested commutators.

A spin-adapted theory for high-spin open-shell systems can be formulated using the same strategy: by constructing the cluster operator from singlet excitation operators relative to an open-shell reference determinant. However, for open-shell systems, several complications arise.

First, there is freedom in the choice of the cluster operator, unlike in the closed-shell theory, and cluster operators have been suggested for open-shell systems that generate either nonorthogonal^4–6^ or orthogonal^7–9^ configurations. While all these approaches yield a spin-adapted theory, the requirement of spin completeness, i.e., that the parametrization sufficiently spans the spin space for all included spatial configurations^5^ requires the inclusion of spectator excitations of higher order than the excitation rank of the model. E.g., in coupled cluster singles and doubles (CCSD), triple and quadruple spectator excitations must be included in the cluster operator to obtain spin completeness for arbitrary quantum number *S*.^5,10^

Second, there are non-commuting terms in the cluster operator, caused by the singly occupied orbitals of the reference determinant. In the spin-free formulation, these orbitals appear as both hole and particle indices in the cluster operator. As a result, the spin-adapted open-shell equations become intractable, as the Baker–Campbell–Hausdorff expansion no longer terminates after four nested commutators. This means that (1) spin-adapted open-shell coupled cluster theory is derived and implemented in an automated manner through the use of symbolic computer programs and (2) that the Baker–Campbell–Hausdorff expansion must be truncated at some order or some other approximations must be introduced. This feature of the open-shell coupled cluster equations has therefore motivated the development of models^11–16^ using a normal ordered exponential operator.^17^

The challenges of open-shell coupled cluster theory that arise because of non-commuting terms in the cluster operator also occur in multireference approaches where the cluster operator is defined relative to a model space (e.g., in Fock space coupled cluster theory^18^) or a CASSCF state (e.g., in the internally contracted multireference coupled cluster model^19^). Such multireference coupled cluster models can be used for open-shell species, in both the high- and low-spin cases. However, a spin-adapted treatment still relies on parametrization in terms of singlet excitation operators.

The models mentioned so far obtain a spin-adapted formulation through the use of singlet (spin-free) excitation operators. A different approach was suggested by Heckert et al.,^20^ who formulated a spin-adapted theory where the cluster amplitudes are determined by solving simultaneously a non-redundant set of projected Schrödinger and spin equations. Heckert et al. define the cluster operator in terms of excitations that generate configuration state functions, which are also used as a projection space when they solve the equations. Hence, as in the spin-adapted theories formulated on the molecular orbital basis, their amplitudes are spin-free, and their formulation in terms of configuration state functions ensures spin completeness. While their approach requires the inclusion of excitations of rank three in the cluster operator in their spin-adapted CCSD model, their approach is fundamentally formulated in the spin–orbital basis, and therefore, there are no non-commuting terms in their cluster operator. The problem with spin contamination in unrestricted coupled cluster theory, and the major challenge evident in the formulation of spin-adapted theories, has motivated the development of models that aims to partially enforce the spin properties of the wave function.^21–25^

Coupled cluster theory—in the closed-shell spin-adapted formulation and in the unrestricted spin-orbital formulation—is an important tool for the calculation of excitation energies. Excitation energies are obtained with linear response^26,27^ theory or the equation-of-motion^28–31^ approach. As is evident from the discussion above, significant efforts have gone into the formulation of a spin-adapted coupled cluster theory of the ground-state wave function of high-spin open-shell systems. For excited states, spin-adapted methods are scarce. Notably, the spin-restricted coupled cluster model by Szalay and Gauss have been extended to the calculation of excited states.^25,32,33^

In addition to excitation energies, the equation-of-motion framework offers a way to describe ionized or electron attached wave functions (e.g., with $S=\frac{1}{2}$ ), even in a spin-adapted closed-shell implementation of the theory. The approach was introduced by Stanton and Gauss,^34^ who included a non-interacting virtual (occupied) orbital in their calculation and ensured that an electron is excited into (out of) that orbital in the excited-state calculation. With this approach, both the ground and excited states of an anionic or cationic system are available on equal footing, starting from the neutral ground state. This approach is simple to implement through projections during the diagonalization of the similarity-transformed Hamiltonian. However, forcing an electron into, or out of, the non-interacting orbital reduces the parametric freedom in the EOM calculation and higher orders of coupled cluster theory are sometimes needed to achieve accuracy comparable to that of standard valence excited states.

Previously, we have presented a proof-of-concept implementation of the entanglement coupled cluster singles and doubles (ECCSD) model for the ground state of doublet systems.^35^ With entanglement coupled cluster theory, we describe high-spin open-shell systems in a spin-adapted manner. For doublet systems, a non-interacting orbital is mixed with the singly occupied molecular orbital of the system. A closed-shell reference determinant for coupled cluster theory is constructed in this mixed orbital basis, and through the use of a projection operator, we ensure that the molecule has the correct electron number and spin state. Here, we present an $O\left(N^{6}\right)$ -scaling implementation of the entanglement coupled cluster singles and doubles model for both ground and excited states of doublet molecular systems.

## Theory

We consider a doublet molecular system, with molecular orbitals {ψ_*p*_} from a restricted open-shell Hartree–Fock (ROHF) calculation, and include in our calculation a non-interacting bath orbital $\psi_{B}$ . The non-relativistic molecular electronic Hamiltonian is given by

1 H=∑pqhpqEpq+12∑pqrsgpqrs(EpqErs−δqrEps)+hBEBB+12gB(EBBEBB−EBB),

where

2 $$E_{pq}=a_{p\alpha }^{†}a_{q\alpha }+a_{p\beta }^{†}a_{q\beta }=E_{pq}^{\alpha }+E_{pq}^{\beta }$$

is a singlet excitation operator, and where *h*_*pq*_ and *g*_*pqrs*_ are the one- and two-electron integrals (the latter in Mulliken ordering). The summations in eq 1 are restricted to the molecular orbitals, while $h_{B}$ and $g_{B}$ define the one- and two-electron interactions within the non-interacting orbital.

We mix the non-interacting orbital with the singly occupied molecular orbital (SOMO) of the molecular doublet $\psi_{S}$ ,

3 $$\psi_{I}=\psi_{S}\cos\theta -\psi_{B}\sin\theta$$

4 $$\psi_{A}=\psi_{S}\sin\theta +\psi_{B}\cos\theta$$

and thereby obtain a new (mixed) orbital basis, defined by the mixing angle θ. In this work, $\theta =\frac{\pi }{4}$ . We refer the reader to ref 35 for a discussion on this parameter. This basis includes the orbitals of the original basis, except for the orbitals $\psi_{S}$ and $\psi_{B}$ , which have been replaced by ψ_*I*_ and ψ_*A*_.

From now on, we let *i*, *j*, and *k* denote the doubly occupied orbitals of the molecular doublet, *a*, *b*, and *c* denote the virtual orbitals of the molecular doublet, and *p*, *q*, *r*, and *s* denote orbitals, in general. The indices *I* and *A* are the orbitals resulting from mixing $\psi_{S}$ and $\psi_{B}$ .

In this mixed orbital basis, we construct a closed-shell reference according to

5 $$|R\rangle =a_{I\alpha }^{†}a_{I\beta }^{†}\prod_{i}a_{i\alpha }^{†}a_{i\beta }^{†}|vac\rangle$$

which will have one more electron compared to the molecular doublet of interest.

For a molecular doublet with a single electron, we obtain the closed-shell mixed orbital reference

6 $$|R\rangle =a_{I\alpha }^{†}a_{I\beta }^{†}|vac\rangle$$

which, expressed in the original basis

7 |R⟩=(cos2⁡θaSα†aSβ†+sin2⁡θaBα†aBβ†−cos⁡θsin⁡θ(aSα†aBβ†−aSβ†aBα†))|vac⟩,

is seen to be the linear combination of the three singlet states obtained by placing two electrons in the two orbitals $\psi_{S}$ and $\psi_{B}.$ In order to recover the determinant with a single electron of α spin in $\psi_{S}$ , we may act on |R⟩ with the projection operator

8 $$P=n_{B}^{\beta }\left(1-n_{B}^{\alpha }\right)=P^{\beta }\left(1-P^{\alpha }\right)=P^{\beta }Q^{\alpha }$$

where $P^{\sigma }=n_{B}^{\sigma }=E_{BB}^{\sigma }$ counts the number of σ electrons in $\psi_{B}$

9 $$P^{\beta }Q^{\alpha }|R\rangle =a_{S\alpha }^{†}a_{B\beta }^{†}|vac\rangle \left(-\cos\theta \sin\theta \right)$$

The projection operator satisfies

10 $$P^{†}=P,,P^{2}=P$$

and commutes with the Hamiltonian in eq 1.

We define the entanglement coupled cluster wave function

11 |ECC⟩=Pexp(T)|R⟩

where the cluster operator *T* is defined as in spin-adapted closed-shell coupled cluster theory^1^ with respect to the mixed orbital basis. We note that higher spin quantum numbers *S* can be considered by adding additional bath orbitals and modifying the projection operator, as outlined in ref 35 for the high-spin triplet.

In the mixed orbital basis, the Hamiltonian becomes

$$H=\underset{pq}{\sum }{\mathrm{h̃}}_{pq}E_{pq}+\frac{1}{2}\underset{pqrs}{\sum }{\mathrm{g̃}}_{pqrs}\left(E_{pq}E_{rs}-\delta_{rq}E_{ps}\right)$$ 12

where ${\mathrm{h̃}}_{pq}$ and ${\mathrm{g̃}}_{pqrs}$ are transformed to the mixed orbital basis, and the summations are over all orbitals. The number operator, used to define P , is

13 $$n_{B}^{\sigma }=\sin^{2}\theta E_{II}^{\sigma }+\cos^{2}\theta E_{AA}^{\sigma }-\cos\theta \sin\theta \left(E_{IA}^{\sigma }+E_{AI}^{\sigma }\right),$$

in the mixed orbital basis.

By multiplying the Schrödinger equation from the left with exp­(−*T*) and projecting onto the vectors {⟨R|,⟨μ|}, we obtain the equations for the ground-state energy and amplitudes

14 $$\left\langle R\left|\mathrm{P̅}\mathrm{H̅}\right|R\right\rangle =E_{0}\left\langle R\left|\mathrm{P̅}\right|R\right\rangle$$

15 $$\Omega_{\mu }=\Omega_{\mu }^{S}E_{0}$$

where

16 $$\Omega_{\mu }=\left\langle \mu \left|\mathrm{P̅}\mathrm{H̅}\right|R\right\rangle$$

17 $$\Omega_{\mu }^{S}=\left\langle \mu \left|\mathrm{P̅}\right|R\right\rangle$$

Here, we have used [H,P]=0 and introduced the notation X̅=exp(−T)Xexp(T) . The equations can be viewed as a change in the projection manifold compared to the standard closed-shell coupled cluster equations.

Entanglement coupled cluster excited states can be obtained within the equation-of-motion framework. We obtain the generalized eigenvalue equations

18 $${\mathrm{H̅}R}^{k}=E_{k}{\mathrm{S̅}R}^{k}$$

where

19 $$\mathrm{H̅}=\left(\begin{array}{ll} \left\langle R\left|\mathrm{P̅}\mathrm{H̅}\right|R\right\rangle & \eta^{T}\\ \Omega & J \end{array}\right)$$

and

$$\mathrm{S̅}=\left(\begin{array}{ll} \left\langle R\left|\mathrm{P̅}\right|R\right\rangle & {{(\eta }^{S})}^{T}\\ \Omega^{S} & J^{S} \end{array}\right)$$ 20

with

ην=⟨R|P̅H̅|ν⟩Jμν=⟨μ|P̅H̅|ν⟩ηνS=⟨R|P̅|ν⟩JμνS=⟨μ|P̅|ν⟩. 21

The lowest generalized eigenvalue is the ground-state energy and the remaining eigenvalues are excited-state energies.

While the ECC model is spin-adapted, it is not spin-complete; i.e., for all spatial configurations that are included in the parametrization, the appropriate spin space is not fully spanned. Obtaining a spin-complete formulation of coupled cluster theory for doublet molecular systems at the singles and doubles level of theory generally requires the inclusion of triple spectator excitations in the parametrization.^5,10,20^ In ECC for doublet states, including triple excitations of the form *E*_*bj*_ *E*_*Ai*_ *E*_*aI*_ in the parametrization will resolve the issue of spin-completeness. Though the computational scaling of the approach will not change by including these triple excitations, the complexity of the equations will significantly increase.

To evaluate the ECC equations, we have first evaluated the projection manifold {⟨μ|P̅} , see ref 35. From eq 13, we see that {⟨μ|P̅} includes excited determinants of excitation rank *n* + 2 where *n* is the order of the truncation of the cluster operator (i.e., the maximal excitation order of the vectors {⟨μ|}). Therefore, in ECCSD, there are contributions to {⟨μ|P̅} from singly, doubly, triply, and quadruply excited determinants.

For the ground state, eqs 15 and 14, a standard spin-adapted closed-shell coupled cluster implementation can be exploited to obtain all contributions to {⟨μ|P̅} that are of excitation order 1 or 2. Terms arising from the reference contributions to {⟨μ|P̅} are trivial to implement. Finally, the terms arising from projection of the Schrödinger equation on the triples’ and quadruples’ contributions to {⟨μ|P̅}

22 $$\langle_{ij}^{ab}|E_{IA}^{\beta },,\langle_{i}^{a}|E_{IA}^{\alpha }E_{IA}^{\beta },,\langle_{i}^{a}|E_{jb}^{\alpha }E_{IA}^{\beta },,\langle_{ij}^{ab}|E_{IA}^{\beta }E_{IA}^{\alpha }$$

must be considered. We note that these contributions do not add terms which scale more steeply than $O\left(N^{6}\right)$ , since they maximally include two free (unrestricted) occupied and virtual indices. These terms are implemented by use of automatic equation and code generation provided by the Julia package SpinAdaptedSecondQuantization^36^ (SASQ). The autogenerated terms have been compared to a previous proof-of-concept implementation, presented in ref 35. We refer the reader to Appendix C of ref 35 for additional implementation details for the ECCSD ground state.

The EOM equations are obtained by evaluating the linear transformation by the similarity-transformed Hamiltonian

23 $$\rho_{\eta }=\left\langle \eta \left|\mathrm{H̅}\right|\nu \right\rangle c_{\nu }$$

and the linear transformation by the metric

24 $$\rho_{\eta }^{S}=\left\langle \eta |\nu \right\rangle c_{\nu }$$

Here, the ⟨η| vectors are those entering {⟨R|P̅,⟨μ|P̅} . The contributions to {⟨R|P̅,⟨μ|P̅} that are singly and doubly excited determinants can be partially extracted from a spin-adapted closed-shell adaptation of EOM coupled cluster. However, in this implementation, all equations for the excited states have been implemented using the automatic code generator.

## Results and Discussion

The ECCSD ground and excited states have been implemented in a development version of the e^*T*^ program.^37^ The default convergence thresholds of e^*T*^ v1.9.x have been used throughout: the Hartree–Fock equations are solved with a residual threshold (maximum norm) of 10^–7^ a.u., Cholesky decomposition of the electron repulsion integrals is performed with a threshold of 10^–4^ a.u., and the coupled cluster ground and excited state equations are solved to residual thresholds (*l*_2_-norm) of 10^–5^ a.u. and 10^–3^ a.u., respectively. FCI calculations are solved to a residual threshold of 1 × 10^–4^ a.u.

### Comparisons to FCI for the Water Cation

In Table 1, we compare ECCSD and FCI energies for the ground and first excited states of the H_2_O^+^ cation, using the cc-pVDZ basis. The ECCSD energies are all within 0.1 *E*_h_ of the FCI energies, and for the ground state and some of the excited states, the error is on the order of 1 m*E*_h_. Using the ECCSD code, we can obtain the EMP2 energy, which for H_2_O^+^/cc-pVDZ is −75.7096 *E*_h_, giving an error of 97.2 m*E*_h_ compared to FCI.

**Table 1:** Total Energies of the First Eight States of H_2_O^+^ Calculated with FCI, EOM-ECCSD (ECCSD), IP-EOM-CCSD (CCSD), and EOM-ROHF-UCCSD (ROHF-UCCSD)^a^

FCI	ECCSD	Char.	|*c*_*S*,max_|^2^	|*c*_*D*,max_|^2^	$\frac{\left|R_{1}\right|}{\left|R\right|}$	CCSD	ROHF-UCCSD
–75.8069	–75.8043 (2.6)					–75.8093 (2.4)	–75.8037 (3.2)
–75.7329	–75.7302 (2.7)	S	0.95	0.001	0.99	–75.7349 (2.0)	–75.7303 (2.6)
–75.5582	–75.5554 (2.8)	S	0.94	0.002	0.99	–75.5573 (0.9)	–75.5554 (2.8)
–75.2872	–75.2743 (12.9)	SD	0.59	0.19	0.97	–75.0714 (215.8)	–75.3182 (31.0)
–75.2803	–75.2780 (2.3)	S	0.83	0.04	0.97	–75.0142 (266.1)	–75.2769 (3.4)
–75.2290	–75.1451 (83.9)	SD	0.47	0.40	0.37	–74.9953 (233.7)	–75.2391 (10.1)
–75.2168	–75.1988 (18.0)	SD	0.56	0.23	0.97	–74.9806 (236.2)	–75.2074 (9.4)
–75.1963	–75.1923 (4.0)	S	0.81	0.04	0.97	–74.9734 (222.9)	–75.1101 (86.2)
MAE [m*E*_h_]	16.2					147.5	18.6

a The EOM-ROHF-UCCSD are obtained with the CFOUR^33^ program. Energies are in *E*_h_. Absolute energy differences are given in parentheses in m*E*_h_. The ECCSD states are assigned to the FCI states based on a comparison of the ECCSD excitation vectors and the dominant configurations in the FCI state. To evaluate the character of the states, we consider the weight of the most important singly and doubly excited determinants contributing to the FCI state, |*c*_*S*,max_|^2^ and |*c*_*D*,max_|^2^. We can, therefore, characterize the FCI excited states according to whether they are dominated by single excitations of the reference (*S*) or they have significant doubles’ contributions (SD) (with |*c*_*D*,max_|^2^ > 0.1). Finally, we present the ECCSD single excitation vector contribution $\frac{\left|R_{1}\right|}{\left|R\right|}$

Additionally, we present the eight lowest energies obtained in an IP-EOM-CCSD calculation and the ground and excited states from an unrestricted CCSD calculation using an ROHF reference (EOM-ROHF-UCCSD calculations with the CFOUR^33,39^ electronic structure program). For the first three doublet states, the performance of the IP-EOM-CCSD model is similar to ECCSD. However, the errors in the higher excited states are on the order of 100 m*E*_h_. The ROHF-UCCSD energies are similar in quality to ECCSD for all excitation energies, with a mean absolute error of 18.6 m*E*_h_, compared to 16.2 m*E*_h_ with ECCSD.

We have characterized the FCI excited-state vectors according to whether they are dominated by singly excited determinants (S), or whether they also have significant contributions from doubly excited determinants (SD), compared to the ROHF reference determinant with a single α-electron in the SOMO. In particular, configurations with a single β-electron in the SOMO are generated by a double excitation of the reference because it requires an excitation into the vacant mixed orbital, ϕ_*A*_, and out of the occupied mixed orbital, ϕ_*I*_. These *spin-flip* configurations (not to be confused with the spin-flip EOM method^38^) are the ones with significant contributions to the excited FCI vectors with SD character. The imbalance in the description of these states is due to the lack of spin completeness; i.e., for a given spatial configuration, certain spin configurations are missing. As previously noted, specific triple excitations could be included in the cluster operator and (or only) in the EOM parametrization to remove the spin-completeness error.

The errors in the ECCSD energies are below 5 m*E*_h_ for the states of S character and at least an order of magnitude higher for the states with SD character. Except for state 6, all ECCSD excited states satisfy $\frac{\left|R_{1}\right|}{\left|R\right|}\geq 0.97$ . However, the ECCSD states that are assigned to the SD states all have a significant contribution from spin-flip generating double excitations. The ECCSD error increases with increasing weight of doubly excited determinants (measured by the square of the largest CI coefficient for a doubly excited determinant, |*c*_*D*,max_|^2^).

### Satellite Ionizations in Ethylene

In Table 2, we present the lowest excitation energies of the ethylene cation (see the Supporting Information for the molecular geometry) calculated with IP-EOM-CCSDT, IP-EOM-CC3, IP-EOM-CCSD, and EOM-ECCSD using the aug-cc-pVDZ basis set. We also compare to EOM-ROHF-UCCSD calculations with the CFOUR.^33,39^ In the EOM-ROHF-UCCSD calculations, only the *M*_*S*_ quantum number is restricted, and quartet states also appear. We have assigned the EOM-ROHF-UCCSD states according to their spatial symmetry, in addition to the energy. The calculated excited states are characterized according to whether they correspond to (direct) ionization of electrons in lower-lying valence orbitals or whether they are satellites (excited ionized states). It is well established that the IP-EOM-CCSD approach cannot describe satellite states accurately because these excitations effectively are pure double excitations of the closed-shell initial state. Hence, triple excitations in the parametrization (at least) are necessary to model these states through the IP-EOM approach. With EOM-ECCSD, satellites that are strongly dominated by single excitations of the cationic initial state, as is the case for the lowest satellite states of ethylene, can be modeled more accurately. Similar results are found for the aug-cc-pVTZ basis set (Supporting Information).

**Table 2:** Excitations of the Ethylene Cation, as Calculated by IP-EOM-CCSDT (CCSDT), IP-EOM-CC3 (CC3), IP-EOM-CCSD (CCSD), EOM-ECCSD (CCSD), and EOM-ROHF-UCCSD (ROHF-UCCSD) Using the aug-cc-pVDZ Basis Set^a^

CCSDT		CC3		CCSD		ECCSD		ROHF-UCCSD
ω [eV]	Char.	ω [eV]	Char.	ω [eV]	Char.	ω [eV]	Char.	ω [eV]
2.4638	direct	2.4811 (0.0173)	direct	2.4760 (0.0122)	direct	2.4808 (0.0179)	direct	2.5698 (0.1060)
4.1359	direct	4.1419 (0.0060)	direct	4.2114 (0.0755)	direct	4.1879 (0.0520)	direct	4.2289 (0.0930)
5.4399	direct	5.4670 (0.0271)	direct	5.5638 (0.1239)	direct	5.5699 (0.1300)	direct	5.5903 (0.1504)
6.8689	satellite	6.9547 (0.0858)	satellite	9.4264 (2.5575)	satellite	6.7310 (0.1379)	satellite	6.7639 (0.1050)
7.8140	satellite	8.1158 (0.3018)	satellite	10.7446 (2.9306)	satellite	8.0787 (0.2647)	satellite	7.3298 (0.4842)
8.6429	direct	8.6710 (0.0281)	direct	8.9111 (0.2682)	direct	8.8686 (0.2257)	direct	8.6010 (0.0419)
9.9157	satellite	10.0948 (0.1791)	satellite	13.2326 (3.3169)	satellite	10.0136 (0.0979)	satellite	9.2632 (0.6525)

a The states are characterized according to whether they are satellites, i.e., an ionization accompanied by an excitation, or if they are (direct) ionizations from lower lying valence orbitals. Absolute errors compared to IP-EOM-CCSDT are given in parentheses. The ROHF-UCCSD calculations are done with CFOUR^33,39^ electronic structure program.

### Core Excitations in the Benzene Cation

Finally, we consider the benzene molecule, which undergoes symmetry breaking due to the Jahn–Teller effect upon ionization. The cation has two minima, with lowered symmetry (*D*_2*h*_) compared to the neutral molecule (*D*_6*h*_).^40^ We use the two optimized geometries (elongated and compressed) from the Supporting Information of ref 40. We report the carbon K-edge excitation energies for the two geometries. The core excitations are calculated using the core–valence separation (CVS) approximation,^41,42^ which separates the core excitations from high-lying valence excited states that represent the continuum. In the CVS approximation, core excitations are considered by neglecting any pure valence amplitudes in the excitation vectors. While the CVS approximation is implemented through projection of the full space excitation vectors for ECCSD, CCSD, and CCSDT, only the contributing terms are calculated for the CC3 model.^43^ The CVS-IP-EOM models are implemented by neglecting all elements of the excitation vectors that do not contain an occupied index corresponding to a core orbital and a virtual index corresponding to the non-interacting bath orbital. For these models, the core excitation energies of the cation are obtained by subtracting the valence IP from the core IPs. We present calculations with CVS-IP-EOM-CCSD, CVS-IP-EOM-CC3, and CVS-IP-EOM-CCSDT of the neutral molecule and CVS-EOM-ECCSD of the cation, see Table 3. The cc-pVDZ basis set is used and additional calculations with the aug-cc-pVDZ basis set can be found in the Supporting Information.

**Table 3:** Core Excitations of the Benzene Cation Calculated with CVS-IP-EOM-CCSDT (CCSDT), CVS-IP-EOM-CC3 (CC3), CVS-IP-EOM-CCSD (CCSD), and CVS-EOM-ECCSD (ECCSD) Using the cc-pVDZ Basis Set^a^

elongated				contracted			
CCSDT	CC3	CCSD	ECCSD	CCSDT	CC3	CCSD	ECCSD
283.22	283.14 (0.08)	284.56 (1.34)	284.22 (1.00)	283.21	283.13 (0.08)	284.55 (1.34)	284.05 (0.84)
283.23	283.15 (0.08)	284.58 (1.35)	284.22 (0.99)	283.21	283.14 (0.07)	284.57 (1.36)	284.05 (0.84)
283.24	283.16 (0.08)	284.59 (1.35)	284.25 (1.01)	283.25	283.17 (0.08)	284.57 (1.32)	284.58 (1.33)
283.26	283.18 (0.08)	284.61 (1.35)	284.25 (0.99)	283.26	283.18 (0.08)	284.60 (1.34)	284.58 (1.32)
283.29	283.21 (0.08)	284.62 (1.33)	284.70 (1.42)	283.28	283.20 (0.08)	284.62 (1.34)	284.62 (1.34)
283.29	283.22 (0.06)	284.63 (1.34)	284.71 (0.31)	283.29	283.21 (0.08)	284.62 (1.33)	284.62 (1.33)
289.06	289.70 (0.64)	297.83 (8.77)	288.75 (0.35)	289.01	289.56 (0.55)	297.90 (8.89)	288.93 (0.08)
289.10	289.71 (0.61)	297.83 (8.73)	288.75 (0.34)	289.01	289.60 (0.59)	297.90 (8.89)	288.93 (0.08)
289.11	289.72 (0.61)	298.10 (8.99)	289.45 (0.35)	289.02	289.62 (0.60)	297.90 (8.88)	288.95 (0.07)
289.11	289.72 (0.61)	298.10 (8.99)	289.46 (0.35)	289.02	289.62 (0.60)	297.90 (8.88)	288.95 (0.07)
289.11	289.72 (0.61)	298.10 (8.99)	289.46 (0.35)	289.29	289.95 (0.66)	298.24 (8.95)	289.77 (0.48)
289.53	289.72 (0.19)	298.10 (8.57)	289.46 (0.07)	289.29	289.95 (0.66)	298.24 (8.95)	289.77 (0.48)

a All energies are given in eV. Absolute errors compared to CCSDT are given in parentheses.

From these calculations, we observe that while CVS-IP-EOM-CCSD provides adequate results for the first six core excitations, the next six excitations have errors of almost 10 eV compared to the CVS-IP-EOM-CCSDT calculations. These constitute satellite states of the core-ionized molecule. For CVS-IP-EOM-CCSD, large errors arise because of the low degree of parametric freedom resulting from projecting out all excitations that do constitute a core ionization. To accurately describe such states with a closed-shell coupled cluster implementation, triple- or higher-order excitations must be included in the parametrization. For the CVS-EOM-ECCSD model, the core excitation energies of the benzene cation are found to have absolute errors well within 2 eV compared to CVS-EOM-IP-CCSDT, and we note that the CVS-EOM-ECCSD model outperforms CVS-IP-EOM-CC3 for the satellite states. This is likely also because of the reduction in parametric freedom in CVS-IP-EOM-CC3, although to a lesser extent than for CVS-IP-EOM-CCSD.

## Conclusions

We have presented the extension of the entanglement coupled cluster singles and doubles (ECCSD) model to the spin-adapted excited states of doublet systems through the equation-of-motion (EOM) formulation. For states that are dominated by single substitutions compared to the ROHF reference determinant, the accuracy is comparable to that of spin-adapted closed-shell CCSD, when the excitation is dominated by single excitations of the reference. While the model is spin-adapted, the parametrization is not spin-complete. The addition of triple excitations in the parametrization that includes an excitation into and out of the mixed orbitals (*E*_*bj*_ *E*_*Ai*_ *E*_*aI*_) could improve the accuracy for states dominated by configurations where the singly occupied orbital of the high-spin reference determinant is occupied by a single β-electron.

We also demonstrated that the EOM-ECCSD method can be used in situations where IP-EOM-CCSD cannot be trusted.

## Supplementary Material



## Notes

Geometries in the xyz format for water and ethylene, excitation energies of the ethylene cation with the aug-cc-pVTZ basis set, summary of the symmetry analysis for the ethylene excited states, and core excitation energies for the two benzene cation geometries with the aug-cc-pVDZ basis set (PDF)
