# Supplementary material for: Excitation Energies from the Entanglement Coupled Cluster Model for Doublets
Source: J Phys Chem A. 2025 Jul 21;129(30):6911–7. doi: 10.1021/acs.jpca.5c01190 (PMC12319896; doi:10.1021/acs.jpca.5c01190)
Supplement: Supplementary file 1 [file jp5c01190_si_001.pdf]

Supporting Information for Excitation Energies from the Entanglement Coupled Cluster Model for Doublets

Sarai Dery Folkestad,* Kristine Lauvstad Kruken, and Henrik Koch

Department of Chemistry, Norwegian University of Science and Technology, NTNU, 7491 Trondheim, Norway

E-mail: sarai.d.folkestad@ntnu.no

Water cation

Geometry (xyz-format)

3

O	0.000000000000	0.000000000000	−0.004762594898
H	0.000000000000	0.801842648150	−0.560344690289
H	0.000000000000	−0.801842648150	−0.560344690289

Ethylene cation

Geometry (xyz-format)

6

C	−3.291853771982	1.405035096385	0.000000000007
C	−1.946455532139	1.490358819554	0.000000000010
H	−3.878440650754	1.621084781667	−0.897932299432
H	−3.846447584390	1.116612947188	0.897932299409
H	−1.391861719715	1.778780968693	−0.897932299403
H	−1.359868653320	1.274309134214	0.897932299408

Results aug-cc-pVTZ

Table S1: Excitations of the ethylene cation, as calculated by IP-EOM-CC3, IP-EOM-CCSD and ECCSD, using the aug-cc-pVTZ basis set. The states are characterized according to whether they are shake-ups, i.e., an ionization accompanied by an excitation, or if they are (direct) ionizations from lower lying valence orbitals.

IP-EOM-CC3		IP-EOM-CCSD		ECCSD	
ω[eV]	Char.	ω[eV]	Char.	ω[eV]	Char.
2.4636	direct	2.4667	direct	2.4835	direct
4.1266	direct	4.2210	direct	4.2051	direct
5.4306	direct	5.5472	direct	5.5599	direct
7.0012	shake-up	9.6606	shake-up	6.6929	shake-up
8.1120	shake-up	10.9667	shake-up	8.0802	shake-up
8.6495	direct	8.9016	direct	8.8731	direct
10.1128	shake-up	13.5060	shake-up	10.0154	shake-up

Symmetry analysis

Table S2: Excitation energies of the ethylene cation, calculated at the IP-EOM-CCSDT/aug-cc-pVDZ level of theory and the irreducible representations of D_{2h} spanned by the states. The symmetry analysis is performed relative to a coordinate system where the molecule lies in the yz-plane and the z-axis is along the carbon-carbon bond. The ground state spans the B_{3u} irreducible representation.

State	Energy [eV]	Symmetry species
1	2.4638	B_{3g}
2	4.1359	A_g
3	5.4399	B_{2u}
4	6.8689	B_{2g}
5	7.8140	B_{2u}
6	8.6429	B_{1u}
7	9.9157	B_{1u}

Benzene cation

Table S3: Core excitations of the benzene cation calculated with CVS-IP-EOM-CC3 (CC3), CVS-IP-EOM-CCSD (CCSD), and CVS-EOM-ECCSD (ECCSD) using the aug-cc-pVDZ basis set. All energies are given in eV.

Elongated			Compressed		
CC3	CCSD	ECCSD	CC3	CCSD	ECCSD
282.95	284.41	284.06	282.94	284.40	283.89
282.96	284.42	284.06	282.95	284.42	283.90
282.97	284.43	284.09	282.97	284.42	284.41
282.99	284.46	284.09	282.99	284.45	284.42
283.01	284.46	284.53	283.01	284.46	284.45
283.02	284.48	284.53	283.01	284.47	284.45
289.50	297.79	288.55	289.41	297.87	288.74
289.50	297.79	288.55	289.41	297.87	288.74
289.52	298.07	289.27	289.42	297.87	288.75
289.53	298.07	289.28	289.42	297.87	288.76
289.54	298.07	289.28	289.75	298.21	289.60
289.54	298.07	289.29	289.75	298.21	289.60
